# Giant Enhancement of Electron–Phonon Coupling in Dimensionality‐Controlled SrRuO_3_ Heterostructures

**DOI:** 10.1002/advs.202300012

**Published:** 2023-04-13

**Authors:** In Hyeok Choi, Seung Gyo Jeong, Taewon Min, Jaekwang Lee, Woo Seok Choi, Jong Seok Lee

**Affiliations:** ^1^ Department of Physics and Photon Science Gwangju Institute of Science and Technology (GIST) Gwangju 61005 Republic of Korea; ^2^ Department of Physics Sungkyunkwan University Suwon 16419 Republic of Korea; ^3^ Department of Physics Pusan National University Busan 46241 Republic of Korea

**Keywords:** artificial heterostructures, electron–phonon coupling, SrRuO_3_, superlattice

## Abstract

Electrons in crystals interact closely with quantized lattice degree of freedom, determining fundamental electrodynamic behaviors and versatile correlated functionalities. However, the strength of the electron–phonon interaction is so far determined as an intrinsic value of a given material, restricting the development of potential electronic and phononic applications employing the tunable coupling strength. Here, it is demonstrated that the electron–phonon coupling in SrRuO_3_ can be largely controlled by multiple intuitive tuning knobs available in synthetic crystals. The coupling strength of quasi‐2D SrRuO_3_ is enhanced by ≈300‐fold compared with that of bulk SrRuO_3_. This enormous enhancement is attributed to the non‐local nature of the electron–phonon coupling within the well‐defined synthetic atomic network, which becomes dominant in the limit of the 2D electronic state. These results provide valuable opportunities for engineering the electron–phonon coupling, leading to a deeper understanding of the strongly coupled charge and lattice dynamics in quantum materials.

## Graphical abstract

The control of electron–phonon coupling is essential for enhancing the performance of future nanoscale electronic and phononic devices. Here it is demonstrated that the electron–phonon coupling in SrRuO_3_ can be largely controlled by multiple intuitive tuning knobs available in synthetic crystals.

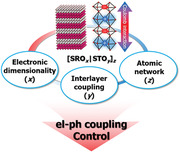

## 1 Introduction

The interaction between electrons and quantized lattice degree of freedom, that is, the electron–phonon (el–ph) coupling, determines the fundamental electrodynamics in crystals, serving as one of the major scattering channels of electronic carriers. It is often responsible for electronic phase transitions between states of matter, such as insulators, metals, and superconductors,^[^ ^7^, ^8^ ^]^ and is essential for developing future nanoscale electronic and phononic devices with thermoelectric, photovoltaic, and thermal functionalities.^[^ ^10^, ^11^ ^]^ For example, the el–ph coupling results in a significant increase in the Seebeck coefficient of thermoelectric nanodevice,^[^ ^12^, ^13^ ^]^ and the formation of a large polaron facilitates the formation of long‐lived high‐temperature hot carriers, which is advantageous for solar cell device application.^[^ ^14^ ^]^ These examples stimulate idea that customization of the el–ph coupling constant (*G*_ep_), if possible, would revolutionize the electronic device concept by providing an additional tuning knob for the correlated charge carriers. However, natural bulk materials have limitations in modulating *G*_ep_ because the electronic and phononic behaviors with their correlation are innately determined by their crystal structures.

One conventional way to control *G*_ep_ is to adjust the doping concentration of the material.^[^ ^15^, ^16^, ^17^, ^18^, ^19^, ^20^ ^]^ However, doping‐induced disorder suppresses both the metallic behavior and phonon dynamics of the system, hampering a selective and systematic control of *G*_ep_. On the other hand, interface engineering has been a promising approach to tuning *G*_ep_, and a fivefold enhancement has been reported by reducing the thickness of a metal film.^[^ ^1^ ^]^ In a graphene/metal heterostructure, *G*_ep_ is enhanced by ≈45 times owing to the contribution of interface phonons or intercalated phonons. In FeSe/SrTiO_3_ (STO) interfaces, ferroelectric optical phonons in STO raise *G*_ep_, leading to a large increase in the superconducting transition temperature.^[^ ^21^, ^22^, ^23^, ^24^ ^]^ Another promising approach to modulate the el–ph coupling is to build superlattices with atomic‐scale precision. In particular, the realization of a well‐defined atomic network via strong covalent or ionic bonding provides an ideal testbed. For example, Nb:SrTiO_3_/SrTiO_3_ superlattices (SLs) and LaAlO_3_/LaNiO_3_/LaAlO_3_ heterostructures exhibit usually large thermopower stemming from the enhanced electron–phonon coupling at the low dimension.^[^ ^10^, ^25^ ^]^ Furthermore, artificially controlled YBa_2_Cu_3_O_7_/La_2/3_Ca_1/3_MnO_3_ SLs facilitated long‐range Coulomb interactions that transfer the el–ph coupling over several tens of nanometers.^[^ ^26^ ^]^ In many cases, however, the control parameters for manipulating *G*_ep_ have been largely limited to a single‐film thickness, and the variation or enhancement of *G*_ep_ has not been significant.^[^ ^5^, ^27^, ^28^ ^]^

**Figure 1:**
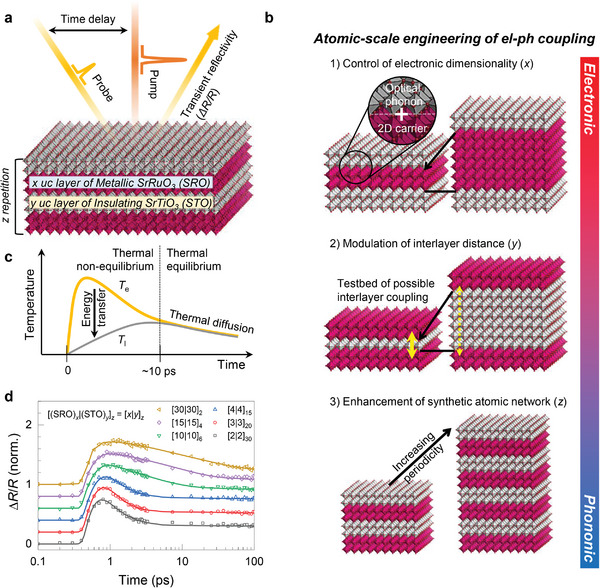
Atomic‐scale engineering of *G*_ep_ in deliberately designed SRO heterostructures. a) Schematic illustration of an optical pump‐probe experiment to characterize *G*_ep_ of atomically controlled [*x*|*y*]*_z_* SLs. b) Schematic representation of our strategy for largely increasing *G*_ep_ of artificial SRO crystal via atomic‐scale precision heterostructuring. c) Schematic diagram of thermal relaxation processes of the photo‐excited hot carriers as a function of time *t*. d) *t*‐dependent Δ*R*/*R* obtained for SRO/STO SLs with various periodicities.

In this study, we demonstrate a giant enhancement of *G*_ep_ in dimensionality‐controlled SrRuO_3_/SrTiO_3_ (SRO/STO) SLs. We compared SRO single‐films and SLs composed of *x* unit cell (uc) layers of correlated metallic SRO and *y* uc layer of quantum paraelectric STO with *z* repetitions (**Figure** **1**a, see Experimental Section). *G*_ep_ could be largely modulated by deliberately adjusting *x*, *y*, and *z*. Figure 1b shows the key features of the proposed strategy. 1) A smaller *x* reduces the electronic dimensionality of SRO,^[^ ^29^, ^30^ ^]^ leading to stronger coupling of the 2D carrier with phonons. 2) Having established the low‐dimensionality‐induced *G*_ep_ enhancement, modulation of *y* provides us with an additional tuning knob of the interlayer coupling strength between the SRO layers. 3) Finally, upon increasing *z*, the synthetic atomic network of the SL becomes better defined with increasing periodicity; hence, the *c*‐axis (out‐of‐plane direction) polarized phonon further improves *G*_ep_. With appropriate controls of *x*, *y*, and *z*, *G*_ep_ of the SRO/STO SL is significantly enhanced by more than 300‐fold compared to that of the thick SRO single‐film. Our findings offer fundamental understanding and exceptional controllability of the el–ph coupling via atomic‐scale heterostructuring of correlated oxides, which will incorporate previously unseen functionalities for upcoming electronic and phononic devices.

## 2 Result and Discussion

To examine *G*_ep_, we exploit an optical pump‐probe technique that traces the energy exchange and relaxation processes of a pump‐induced non‐equilibrium el–ph coupled state. Figure 1c schematically depicts the thermal relaxation processes of photoexcited hot electrons with time‐ (*t*‐) domain electron temperature (*T*_e_) and lattice temperature (*T*_l_). First, the incident photons (pump) are absorbed in the SRO layers and transiently excite non‐thermalized electrons. Second, *T*_e_ increases significantly due to carrier–carrier scattering (hot electron), resulting in a thermal non‐equilibrium state (*T*_e_ ≠ *T*_l_). Third, the energy of electrons is transferred to phonons via the el–ph coupling, which decreases *T*_e_ and increases *T*_l_. Fourth, when *T*_e_ = *T*_l_, that is, thermal equilibrium is reached, both *T*_e_ and *T*_l_ continuously decrease via thermal diffusion. By recording the pump‐induced reflectivity change (Δ*R/R*) as a function of *t* (Figure 1d), we trace and analyze the *T*‐evolutions to determine the physical parameters of the thermal processes. Here, *R* and Δ*R* are the reflectivity obtained without the pump pulse and the relative reflectivity change in the probe pulse induced by the pump pulse, respectively. Δ*R/R* curves are normalized to each peak amplitude and shifted vertically with constant offset. As the thicknesses of SRO and STO layer decrease, Δ*R/R* curves of SRO/STO SLs show a much faster decay. The large variation of thermal relaxation in SRO/STO SLs validates our approach to control the el–ph coupling using the artificial heterostructuring.

**Figure 2:**
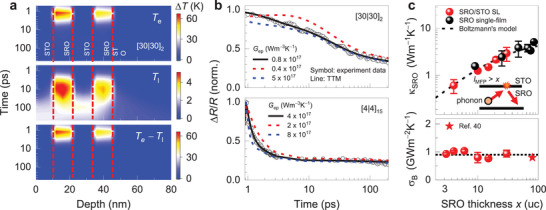
Comparison between TTM simulation and pump‐probe experimental results. a) TTM simulation result of a spatio‐temporal dependent *T*_e_ (top panel), *T*_l_ (middle panel), and *T*_e_–*T*_l_ (bottom panel), respectively, for [30|30]_2_ SL. b) Comparison between experimental and fitting results with three different *G*_ep_ values for two [30|30]_2_ (top panel) and [4|4]_15_ (bottom panel) SLs. c) Summary of *x*‐dependent *κ*_SRO_ (top panel) and *σ*_B_ (bottom panel) obtained from the TTM analysis for the pump‐probe results. When *l*_MFP_ > *x*, the *x*‐dependence of *κ*_SRO_ can be well described by Boltzmann's transport model, as schematically shown in the inset. On the other hand, *σ*_B_ is independent of *x*, and these values are similar to the reference value measured by time‐domain thermoreflectance.^[^ ^4^ ^]^

To quantitatively extract the *G*_ep_ values of SRO heterostructures, we used the two‐temperature model (TTM), which describes both the el–ph thermalization and thermal diffusion processes (**Figure** **2**, see Experimental Section and Sections S3, S4, and S5, Supporting Information, for details). Figure 2a shows the results for [30|30]_2_ SL as an example. The top, middle, and bottom panels represent the spatiotemporal evolution of *T*_e_, *T*_l_, and their difference, respectively, resembling the schematic shown in Figure 1c. In the SRO layer, *T*_e_ and *T*_l_ peak at ≈1 and 10 ps, respectively, following the photoexcitation. After ≈10 ps, the difference between *T*_e_ and *T*_l_ becomes negligible. This thermalization time is related to *G*_ep_ which is proportional to the energy exchange rate between the electrons and phonons. Note that an electron–phonon inelastic scattering is involved in this process. After the electron–phonon thermalization in the SRO layer, *T*_l_ of the STO layer increases owing to the thermal diffusion process, and both the SRO and STO layers reach thermal equilibrium at about 100 ps. After ≈100 ps, both *T*_e_ and *T*_l_ in the SLs slowly decrease via thermal diffusion toward the substrate. Figure 2b and Figure S5, Supporting Information, show that the TTM well describes the experimentally observed Δ*R/R* evolution of the films, providing sufficient sensitivity for obtaining *G*_ep_, thermal conductivity (*κ*), and thermal boundary conductance (*σ*_B_). Indeed, Figure 2b shows that the *G*_ep_ value sensitively determines the TTM fitting results (especially for the fast‐decay region). The top panel of Figure 2c summarizes the *x*‐dependent *κ* of the SRO layer (*κ*_SRO_) and *σ*_B_ between the SRO and STO layers within the heterostructure. When the phonon mean free path (*l*_MFP_) is greater than *x*, the interface scattering of phonons decreases *κ*_SRO_ following the Boltzmann's transport model^[^ ^21^ ^]^ as $\kappa_{\mathrm{SRO}}\;=\;\frac{\kappa_{\mathrm{bulk}}}{1\;+\;(2\beta \;l_{\mathrm{MFP}})/x}$, where *κ*_bulk_ and *β* are the bulk thermal conductivity and thickness‐dependent free variable, respectively, and the latter ranges from 0.67 to 0.71. The experimental results (symbols) are well reproduced by Boltzmann's transport model (dashed line) with *l*_MFP_ = 20 nm and *κ*_bulk_ = 5.5 W m^−1^ K^−1^ in good agreement with the previous results obtained by the conventional *t*‐domain thermo‐reflectance method.^[^ ^31^ ^]^ On the other hand, the bottom panel of Figure 2c shows that *σ*_B_ is nearly independent of *x*, indicating well‐defined SRO/STO interfaces of SLs. These results consistently validate our analyses for characterizing *G*_ep_ values of the SRO heterostructures.

**Figure 3:**
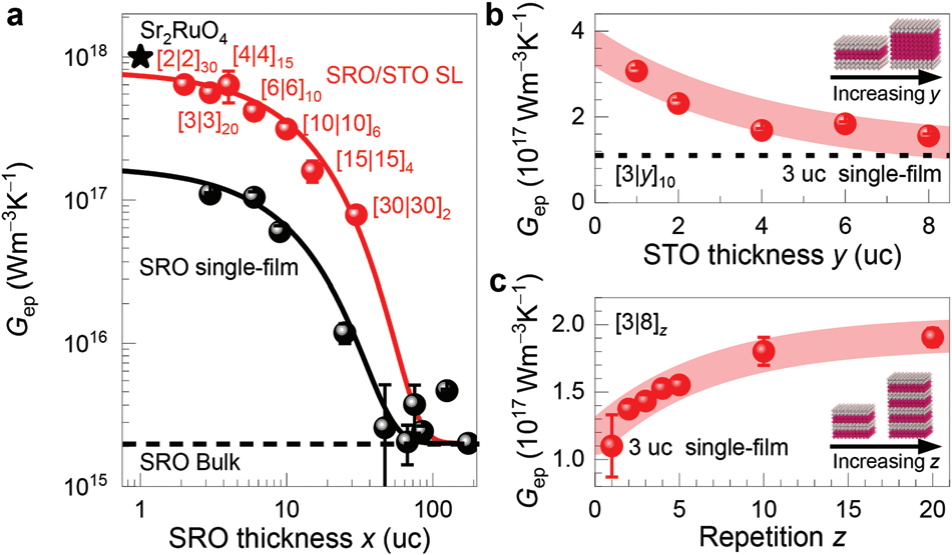
Giant enhancement of *G*_ep_ in atomically designed SRO single‐film and SRO/STO SL. a) *x*‐dependent *G*_ep_ for atomically controlled SRO heterostructures. With a reduction of the SRO dimensionality, *G*_ep_ becomes significantly enhanced in both single‐films (black circles) and SLs (red circles) whereas the enhancement is larger in the SLs. Black and red solid lines are simple exponential fitting curves. *G*_ep_ of Sr_2_RuO_4_^[^ ^2^ ^]^ (star symbol) is included at the position of a single SRO uc. Modulation of *G*_ep_ with precisely controlled b) *y* and c) *z* of SRO heterostructures, suggesting a possible interlayer coupling of SRO/STO SL similar to that of Sr_2_RuO_4_ layered perovskite.

**Figure** **3** summarizes *G*_ep_ values of the SRO layers, which are largely and systematically modulated via atomic‐scale heterostructuring. Figure 3a shows *x*‐dependent *G*_ep_ of the SRO heterostructures with a giant enhancement of *G*_ep_ at the atomically thin (quasi‐2D) limit. Above a single‐film thickness of 20 uc, *G*_ep_ is estimated to be as low as 10^15^ W m^−3^ K^−1^ similar to bulk SRO.^[^ ^32^ ^]^ As *x* decreases, *G*_ep_ increases significantly to about 10^17^ W m^−3^ K^−1^ for 3 uc of SRO single‐films. More interestingly, *G*_ep_ of the SRO/STO SLs is further enhanced by an order of magnitude greater than that of a single‐film at the same *x*. Such a large enhancement of *G*_ep_ (≈32 500% improvement compared to that of bulk) has never been reported before (**Table** **1**). The SRO thickness‐dependent *G*_ep_ can be described by the phenomenological function *G*_ep_ = *G*_bulk_ + *e^−x^* ^/^ *^c^ G*_0_ with *G*_bulk_ = 2 × 10^15^ W m^−3^ K^−1^. For the best fit, *G*_0_ values are chosen as 6.6 × 10^16^ and 3.6 ×10^17^ W m^−3^ K^−1^ for single‐films and SLs, respectively, and the characteristic parameter *c* is the same as 4 nm for both the single‐film and SL cases. This clearly demonstrates that the dimensionality reduction in both the SRO single‐film and SL causes a large enhancement of *G*_ep_, whereas the periodic structure of SLs leads to further enhanced *G*_ep_ values compared to those of SRO single‐films. The atomic‐scale control of the other geometrical parameters (*y* and *z*) within quasi‐2D SRO SLs (*x* = 3) provides extended controllability of *G*_ep_, representing the distinctive advantages of artificial crystal structures. Figure 3b shows that *G*_ep_ of the [3|*y*]_10_ SLs increases with decreasing *y*, representing the possible contribution of the enhanced interlayer coupling between the quasi‐2D SRO layers within the SLs. Furthermore, as *z* increases, *G*_ep_ monotonically increases to 1.9 × 10^17^ W m^−3^ K^−1^ (for *z* = 20), which is slightly less than twice that of the single film (*z* = 1), owing to the improvement of the synthetic atomic networks (Figure 3c). These results reveal the extremely high controllability of *G*_ep_ by directly manipulating the synthetic crystal with atomic‐scale precision.

**Table 1:** Experimental demonstrations of modulation of el–ph coupling constant *G*_ep_ including the present work on SRO/STO superlattices

Material	Enhancement origin	*G*_ep_ [10^16^ W m^−3^K^−1^]	Enhancement factor	Reference
Au/Ti/Al_2_O_3_	Interface phonon	2.2–11	≈5	[1]
Cu/Si	Interface phonon	4.5–8.8	≈2	[5]
Au/air	Surface plasmon	2.2–80	≈40	[6]
Au/Ag/Pt nano‐shell	Increasing free carrier density	2.2–15	≈8	[9]
Atomically designed SRO/STO superlattice	Dimensionality control + Interlayer Coulomb interaction	0.2–65	≈325	This work

**Figure 4:**
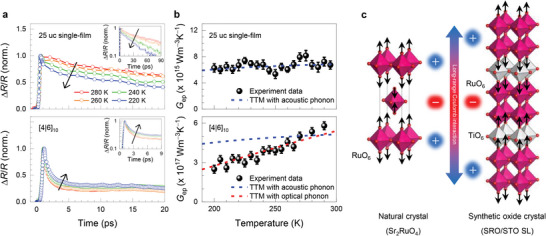
Dimensionality‐controlled el–ph coupling and its temperature dependence for atomically controlled SRO heterostructures. a) *T*‐dependent Δ*R/R* for 25 uc single‐film (top panel) and [4|6]_10_ SL (bottom panel). Inset shows the log scale of Δ*R/R* for different temporal ranges, representing different relaxation time scales. b) *T*‐dependent *G*_ep_ and its comparison with the TTM. Although the fitting results of 25 uc single‐film are well described by considering the acoustic phonon contribution with the Debye temperature 390 K,^[^ ^3^ ^]^ that of [4|6]_10_ SL is described by the contribution of an optical phonon with a resonance at 96 meV. c) Sketch of possible interlayer coupling of SRO/STO SL, similar to that of Sr_2_RuO_4_ layered perovskite.

Let us discuss the possible origin of the unprecedented *G*_ep_ enhancement in SRO heterostructures. One important finding is that the *G*_ep_ enhancement is accompanied by a reduction in the electron density of states at the Fermi level (Figures S3a and S11, Supporting Information) in contrast to conventional metallic systems. Therefore, we pay attention to the modulation of phonon contribution. Because the phonon population is strongly dependent on *T*, we examined *T*‐dependent changes in Δ*R*/*R* and *G*_ep_, as shown in **Figure** **4**a,b, respectively. Figure 4a displays the *T*‐dependent Δ*R*/*R* curves for the 25 uc SRO single‐film (3D, top panel) and the [4|6]_10_ SL (quasi‐2D, bottom panel). Because of the modulated electronic dimensionality of SRO, they exhibit a large difference in relaxation time across all *T* ranges investigated, as discussed previously. More interestingly, the *T*‐dependent relaxation behavior of the SL is opposite to that of the single film. As *T* decreases, relaxation of the single‐film occurs faster, whereas that of the SL becomes slower. The former and latter are typical *T*‐dependent behaviors of the relaxation time when acoustic phonons and optical phonons, respectively, are involved in hot electron energy relaxation.^[^ ^33^, ^34^ ^]^ We quantitatively determine *T*‐dependent *G*_ep_ values, as shown in Figure 4b. With decreasing *T*, *G*_ep_ of both single‐film (black symbol) and SL (red symbol) decreases, which can be naturally attributed to the reduction of the phonon population at a lower *T*. To account for such *T*‐dependences more quantitatively, we simulate the *T*‐dependent *G*_ep_ curves using TTM by considering two different contributions, that is, acoustic and optical phonons for the el–ph energy transfer rate.^[^ ^33^ ^]^ *T*‐dependent *G*_ep_ for the SRO single‐film is well described by the TTM with the acoustic phonon contribution only (top panel). In contrast, for the SL case, the TTM with optical phonons well explains the *T*‐dependent *G*_ep_ (bottom panel). In this analysis, the energy scale of the resonant optical phonon is set to 96 meV, which corresponds to that of the STO polar LO_4_ mode.^[^ ^35^ ^]^ From an angle‐resolved photoemission spectroscopy study,^[^ ^35^ ^]^ Wang et al. claimed that this oxygen vibration mode is strongly coupled with the 2D electron liquid at the STO surface, if any, and a large polaron is formed owing to long‐range el–ph coupling. We also confirmed the Raman excitation at 780 cm^−1^ (96 meV) of the SRO/STO SL using confocal Raman spectroscopy, where the corresponding peak is absent for the cubic STO substrate and thick SRO film (Supporting Information S2). This correspondence strongly supports the existence and influence of polar LO_4_ phonons in SRO/STO SL, whose coupling to quasi‐2D carriers in SRO layers may lead to a giant enhancement of *G*_ep_ for atomically designed SRO/STO SLs.

*G*_ep_ control by varying *y* and *z* within SLs further provides an important clue to the *G*_ep_ enhancement mechanism in quasi‐2D SRO SL. Because there is no significant change in the electronic state of the SRO layer even if *y* is varied down to 1,^[^ ^36^ ^]^ the *G*_ep_ enhancement with a reduction in the interlayer spacing *y* (Figure 3b) suggests a positive correlation with the interlayer interaction between the quasi‐2D SRO layers and *G*_ep_. This implies that the long‐range Coulomb interaction is responsible for the non‐local el–ph coupling and that its spatial extent reaches as far as 3.2 nm of the insulating STO layer thickness (Figure 3b). The enhancement of *G*_ep_ with increasing *z*, as shown in Figure 3c, highlights the role of the *c*‐axis polarized phonon. As *z* increases, the interface density of the SL remains the same, whereas the number of interfaces increases. Because our measurement reflects the average response of the entire probing depth, we conclude that the large *G*_ep_ enhancement in the SLs does not originate from the interface effect. Rather, the *G*_ep_ enhancement with increasing the repetition *z* evidences a possible contribution of the *c*‐axis polarized phonon, which should be better defined in SLs with many repetitions leading to the increase of electron–phonon scattering cross‐section. Also, the large *z* makes an increase of the Madelung‐like potential which also can lead to the *G*_ep_ enhancement as the larger ionic potential change is expected with given ionic displacements of *c*‐axis polarized phonon. Thus, *y*‐ and *z*‐ dependent variations of *G*_ep_ suggest that the el–phonon coupling in quasi‐2D SRO SL is enhanced also by the interlayer interaction mediated by *c*‐axis polarized phonons whereas the dimensionality reduction is having a dominant contribution.

Note that *G*_ep_ value of the quasi‐2D [2|2]_30_ SL is similar to that of the layered perovskite Sr_2_RuO_4_ (star symbol in Figure 3a). Inelastic neutron scattering studies of Sr_2_RuO_4_ have suggested that the optical phonon mode at the Z‐point corresponding to apical oxygen vibration along the *c*‐axis (O*_zz_* mode) is strongly coupled with electrons in quasi‐2D RuO_2_ layers because of weak electric field screening,^[^ ^37^ ^]^ as schematically shown in the left panel of Figure 4c. The apical oxygen in each RuO_2_ layer moves alternatively toward or away from the Ru layer along the out‐of‐plane direction, which is strongly coupled with interlayer charge transfers via unscreened Madelung‐like electrostatic interactions. In the case of the SRO thin film, it undergoes a transition from ferromagnetic‐metal to antiferromagnetic‐insulator accompanying 2D electronic state at 2 uc.^[^ ^30^ ^]^ Hence the decreasing of *x* leads to reduce the electric field screening along the *c*‐axis gradually. Furthermore, zone‐folding effects in SL phonon dispersion give rise to an unexpected correlation between quasi‐2D electrons and phonons away from the original Brillouin center or boundaries.^[^ ^26^ ^]^ As schematically shown in the right panel of Figure 4c, we speculate that the out‐of‐plane breathing mode at the zone‐boundary of the SL can extend to supercell structures, similar to the oxygen vibration O*_zz_* mode of Sr_2_RuO_4_ and the LO_4_ phonon mode at the R‐point of STO. Owing to the 2D confinement effect in the atomically thin SRO layer of SL, the *c*‐axis polarized mode in Figure 4c endows weak electronic screening and can couple with 2D electrons in the SRO layers, as in Sr_2_RuO_4_.

## 3 Conclusion

In summary, we demonstrated a giant enhancement of el–ph coupling in SRO SLs. We controlled the structural parameters of the synthetic oxide SL with atomic‐scale precision, providing an extremely wide tunability of the el–ph coupling. In particular, *G*_ep_ of the quasi‐2D SRO layer increased by more than 300 times compared to that of bulk SRO. The *T*‐dependence of *G*_ep_ reveals that the 2D carrier of the SRO layer is coupled with optical phonons, leading to an unprecedented enhancement in *G*_ep_ for the atomically thin SRO heterostructure. By changing the interlayer distance and atomic networks, we further estimated the possible interlayer coupling between quasi‐2D SRO layers. We note that the further experimental and theoretical investigations are strongly desired for the clearer verification of these observations. Our findings provide fundamental understanding and exceptional controllability of the el–ph coupling via atomic‐scale heterostructuring of correlated oxides, potentially leading to unprecedented functionalities for future electronic and phononic devices.

## 4 Experimental Section

### Atomic‐Scale Heterostructuring

Epitaxial SRO single‐films and SLs grown on a (001)‐oriented single‐crystal STO substrate were prepared using pulsed laser epitaxy.^[^ ^30^, ^36^, ^38^, ^39^, ^40^, ^41^, ^42^ ^]^ Stoichiometric ceramic SRO and STO targets, and a KrF excimer laser (248 nm; IPEX 868, Light Machinery) were utilized. A 1.5 Jcm^−2^ laser fluence and 5 Hz repetition rate were used. For the stoichiometric conditions of both SRO and STO layers, a substrate temperature of 750 °C and an oxygen partial pressure of 100 mTorr were employed. *x* uc of SRO and *y* uc of STO were precisely controlled with *z* repetition to examine the interplay of the crystal lattice and dimensionality to determine the el–ph coupling (1 uc of perovskite oxide layer is ≈0.4 nm). [6|6]_10_ SLs were further synthesized on various substrates, namely, LAO (LaAlO_3_), NGO (NdGaO_3_), LSAT ((La_0.18_Sr_0.82_)(Al_0.59_Ta_0.41_)O_3_), DSO (DyScO_3_), GSO (GdScO_3_), and NSO (NdScO_3_), which endowed the strain from −4% to 2% for investigating possible strain effects. The atomically well‐defined periodicity of superlattices was confirmed using X‐ray reflectivity and *θ*–2*θ* measurements (high‐resolution X‐ray diffractometer of PANalytical X'Pert) (Figure S1b–d, Supporting Information). Atomic force microscopy images further showed a typical step‐terrace structure of the surface, indicating atomically flat surfaces (Figure S1a, Supporting Information).

### Optical Pump–Probe Experiment

An optical pump and an optical probe method were employed to trace the relaxation process of the photo‐excited hot carriers. A normal‐incidence reflection geometry was adopted. For both pump and probe beams with a center wavelength near 800 nm, a transform‐limited pulse duration of ≈80 fs FWHM by chirp pre‐compensation could be obtained. The wavelength of pump laser selectively excited the hot carrier in the SRO layer within the superlattices. The pump laser beam was tightly focused on the sample using a 20× objective lens (N.A. = 0.55) with a fluence of 200 µJ cm^−2^, and generated 5 × 10^20^ cm^−3^ photocarriers that are sufficiently less than a static carrier density^[^ ^30^ ^]^ in SRO layer. The photocarrier generation in STO layers and the substrate can be ignored due to its transparency to the pump laser beam. A photoelastic modulator (HINDS instrument, 100 kHz) was used to modulate the pump beam, and the probe beam monitored the pump‐induced changes in reflectivity with a time delay of up to 300 ps.

### Thermal Relaxation Analysis Based on the Two‐Temperature Model

To obtain a quantitative understanding of the hot electron relaxation process, the experimental results were fitted by considering both the electron–phonon thermalization and thermal diffusion processes. Upon photo‐excitation, the reflectance varied owing to the changes in *T*_e_ and *T*_l_ as Δ*R* = (∂*R*/∂*T*_e_) Δ*T*_e_ + (∂*R*/∂*T*_l_) Δ*T*_l_.^[^ ^33^ ^]^ In obtaining the average temperature for the superlattice, the substrate contribution was excluded by considering its small thermoreflectance coefficient.^[^ ^43^, ^44^ ^]^ In the present condition, Δ*T*_e_ amounted to about 100 K, and the reflectivity change was ≈0.01%. The evolutions of *T*_e_ and *T*_l_ were determined by the energy transfer between the two subsystems as well as by electron and phonon diffusion along the depth (*x*). Because the beam diameter (2 µm) was much larger than the SRO thickness (<50 nm), quasi‐1D thermal conduction along the depth (*a*) can be considered. Accordingly, the following equation should be satisfied

(1) $$C_{e}\left(a\right)\frac{dT_{e}\left(a,\;t\right)}{\mathrm{dt}}=G_{\mathrm{ep}}\left(T_{l}\left(a,\;t\right)-T_{e}\left(a,\;t\right)\right)+\kappa_{e}\frac{d^{2}T_{e}\left(a,\;t\right)}{da^{2}}\;+\;S(a,\;t)$$

(2) $$C_{l}\left(a\right)\frac{dT_{l}\left(a,\;t\right)}{dt}\;=\;G_{\mathrm{ep}}\;\left(T_{e}\left(a,\;t\right)\;-\;T_{l}\left(a,\;t\right)\right)\;+\kappa_{l}\frac{d^{2}T_{l}\left(a,\;t\right)}{da^{2}}$$

here, *C*_*i* = e, l_ and *κ*_*i* = e, l_ are the heat capacity and thermal conductivity, respectively, for the electron (*i* = e) and lattice (*i* = l), and the source term *S*(*a*, *t*) was proportional to the spatiotemporal distribution of the pump laser power.^[^ ^33^ ^]^ In the two‐temperature analysis, the electron–phonon coupling constant *G*_ep_ represented the total energy relaxation rate to both acoustic and optical phonon channels. Considering that the electron temperature increase is smaller than 100 K, *G*_ep_ here was a temperature‐independent constant value.^[^ ^45^ ^]^ *κ*_e_ was set to zero not only for the insulating STO but also for the metallic SRO; as thermalized hot electrons in one SRO layer cannot directly transfer their energy to another conducting SRO layer because of the insulating STO layers in between; the cross‐plane electron thermal diffusion can be safely ignored. Also, the electron specific heat of STO was set to zero due to negligible free carrier density in STO. Therefore, *T*_e_ can be defined in STO, but it was instantly thermalized with lattice. The phonon specific heat was taken from the literatures as *C*_l_ = 2.85 and 2.56 J cm^−3^ K^−1^ for SRO and STO, respectively.^[^ ^31^ ^]^ The electron heat capacity was ignored for the insulating STO, and that for the SRO was estimated from the density of states at the Fermi level for a given SRO thickness (Supporting Information S3). At the bottom and top surfaces, the Neumann boundary condition was considered as $\kappa_{i}\;\frac{dT}{da}=\;0$. At the interface between the SRO and STO including the substrate, the boundary condition with the thermal boundary conductance *σ*_*B*_ was considered as $\kappa_{i}\;\frac{dT}{da}=\sigma_{B}\;\left(T_{2}-T_{1}\right)$. The parameters of *κ*, *G*_ep_, and *σ*_B_ were varied and determined by fitting the experimental results of Δ*R*, and the thickness‐dependences of each parameter are displayed in Figure 2c and Figure 3a, respectively. See the Supporting Information for further details.

## Conflict of Interest

The authors declare no conflict of interest.

## Author Contributions

I.H.C. and S.G.J. contributed equally to this work. S.G.J. and W.S.C. prepared the superlattices and characterized them. I.H.C. and J.S.L. performed the pump‐probe experiments and analyzed the data. T.M. and J.L. performed the DFT calculation. I.H.C., S.G.J., W.S.C., and J.S.L. wrote the manuscript. All of the authors discussed the results and implications, and commented on the manuscript. W.S.C. and J.S.L. supervised the project.

## Supporting information

Supporting Information

## Data Availability

The data that support the findings of this study are available from the corresponding author upon reasonable request.
