# Supplementary material for: Giant Enhancement of Electron–Phonon Coupling in Dimensionality‐Controlled SrRuO3 Heterostructures
Source: Adv Sci (Weinh). 2023 Apr 13;10(16):2300012. doi: 10.1002/advs.202300012 (PMC10238185; doi:10.1002/advs.202300012)
Supplement: Supplementary file 1 — Supporting Information [file ADVS-10-2300012-s001.pdf]

Supplementary information

Giant enhancement of electron-phonon coupling in atomically controlled $SrRuO_3$ heterostructures

In Hyeok Choi[1]*, Seung Gyo Jeong*[2]*, Taewon Min*[3]*, Jaekwang Lee*[3]*, Woo Seok Choi*[2]*, and Jong Seok Lee*[1]

[1]Department of Physics and Photon Science, Gwangju Institute of Science and Technology (GIST), Gwangju 61005, Republic of Korea

[2]Department of Physics, Sungkyunkwan University, Suwon 16419, Republic of Korea

[3]Department of Physics, Pusan National University, Busan 46241, Republic of Korea

S1. Atomically designed SRO/STO SLs grown by pulsed laser epitaxy

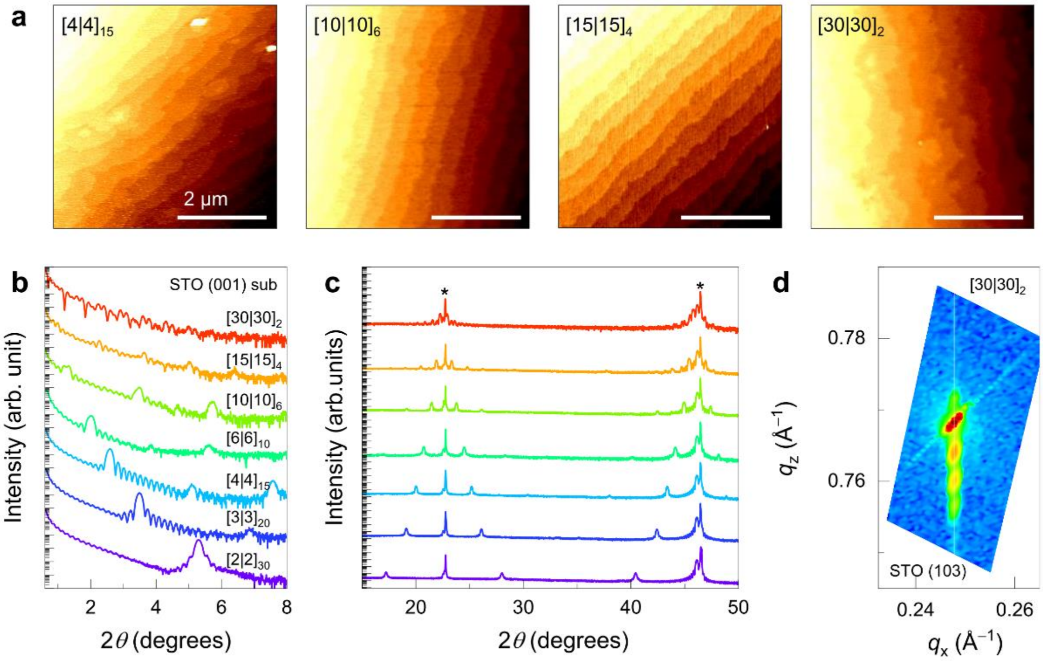

Figure S1. Structural characterization of the atomically designed SRO/STO ($[x|y]_z$) SLs. (a) Atomic force microscopy topographic image for the SRO/STO SLs shows an atomically flat step-terrace structures. (b) X-ray reflectivity and (c) X-ray diffraction θ-2θ scan results confirm the SL Bragg peaks, representing the atomically controlled periodicity of SL. The asterisk (*) denotes the STO substrate peaks. (d) Reciprocal space map of $[30|30]_2$ SL around the STO (103) plane indicates the epitaxial fully strained SL. The vertical solid line is a guide to the eye.

S2. Observation of enhanced phonon modes in SRO/STO SL using confocal Raman spectroscopy

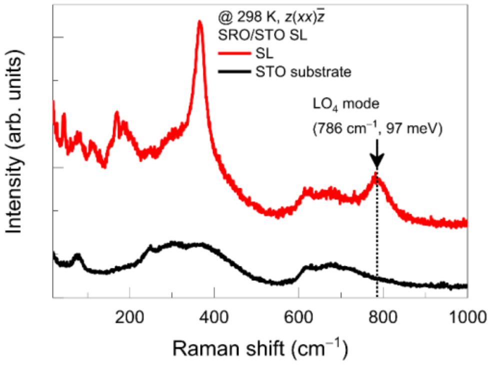

Figure S2. Confocal Raman spectroscopy results of $[6|6]_{50}$ SLs at room temperature with $z(xx)\bar{\mathbf{z}}$ polarization . By controlling the vertical beam position (d), we focus on the SL (d = 0, red line) and STO substrate (d = 20 μm, black line)[1]. More various phonon mode are visible in the Raman spectra of SL compared to that of substrate (more detailed assignments of phonons are in reference[3]). In particular, Raman excitation at ~786 cm^{-1} clearly increase for SL, corresponding to energy scale of LO_4 phonon mode in STO layer.

S3. Parameters used for the two-temperature model (TTM) analysis

In solving the so-called rate equations introduced as Eq. 1 and 2 in the main text, several parameters should be pre-determined. Among them is the electron heat capacity γ_e of the $SrRuO_3$ (SRO) layer. γ_e of the $SrTiO_3$ (STO) layer is ignored because of its insulating nature, and that for SRO is taken into account to be varied depending on the SRO thickness. We perform the density functional theory (DFT) calculation for the SRO system, and estimate γ_e from the density of states at the Fermi level for the given SRO thickness as shown in Fig.S3(a). Our DFT calculation indicates that the SRO layer becomes insulating at the 2 unit-cell (uc) thickness. However, the $[2|6]_{10}$ SRO/STO superlattice (SL) exhibits a metallic character at room temperature[4]. We therefore take γ_e for the 2 uc SRO layer from a linear extrapolation of the thickness-dependent γ_e. By the way, we use the bulk value for the SRO films thicker than 10 uc.

For the thermal conductivity κ, although κ of the SRO layer is taken as a fit parameter in our analysis as demonstrated in Fig. 2c in the main text, we consider that of the STO layer to be pre-determined based on the Boltzmann's transport model given as $\kappa_{\mathrm{SRO}}=\frac{\kappa_{\mathrm{bulk}}}{1+(2\beta\, l_{\mathrm{MFP}})/x}$, where β is a thickness-dependent parameter. Note that this prediction works well in describing the thickness-dependent thermal conductivity of the SRO layer as shown in Fig. 2(c). For the STO layer, the bulk thermal conductivity (κ_{bulk}) and phonon mean free path are 11 $Wm^{-3}K^{-1}$ and 2.35 nm[5,6], respectively, and Fig. S3(b) shows an effective thermal conductivity (κ_{eff}) of the STO layer which is taken as a pre-determined parameter in the simulation. In our previous research[4], our DFT calculation within the GGA+U scheme explains well the metal-insulator transition in SRO/STO SL consistently with experimental observations. Therefore, we believe that our DFT calculation can provide reasonable values of the

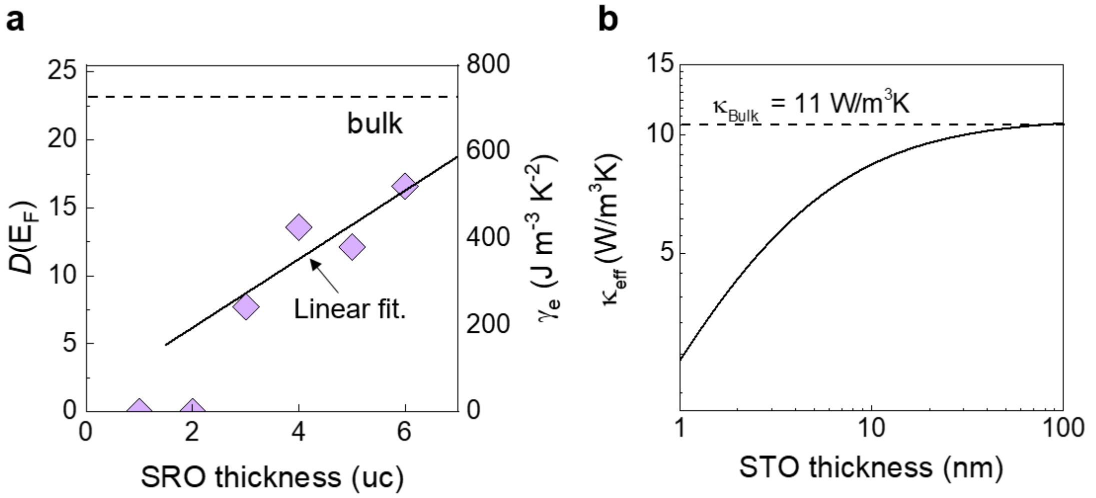

Figure S3. Electron specific heat of the $SrRuO_3$ (SRO) layer and effective thermal conductivity of the $SrTiO_3$ (STO) layer depending on the thickness. (a) Density of states at the Fermi level and electron specific heat of the SRO layer obtained from the density functional theory calculation. (b) Effective thermal conductivity of STO obtained from the Boltzmann's transport model[2].

density of states or the electron specific heat for ruthenates, at least to some extent.

To solve the rate equations (1) and (2) introduced in the main text, we adopt a finite difference method. As depicted in Fig. S4, Eq. S1 and S2 are discretized by using the Euler method and the box method as

$$\begin{pmatrix} T_{e,i}^{j+1} \\ T_{l,i}^{j+1} \end{pmatrix} = \begin{pmatrix} T_{e,i}^{j} \\ T_{l,i}^{j} \end{pmatrix} + G_{ep}\Delta t \begin{pmatrix} -1/C_{e,i} & 1/C_{l,i} \\ 1/C_{l,i} & -1/C_{e,i} \end{pmatrix} \begin{pmatrix} T_{e,i}^{j} \\ T_{l,i}^{j} \end{pmatrix} + \frac{\kappa_i \Delta t}{\Delta a C_{l,i}} \begin{pmatrix} 0 \\ q_{i+0.5}^{j} - q_{i-0.5}^{j} \end{pmatrix} + \begin{pmatrix} S_i^j \\ 0 \end{pmatrix} \quad \text{(S1)}$$

$$q_{i+0.5}^{j} = \frac{1}{\Delta a}\left(T_{l,i+1}^{j} - T_{l,i}^{j}\right) \quad \text{(S2)}$$

$$q_{i-0.5}^{j} = \frac{1}{\Delta a}\left(T_{l,i}^{j} - T_{l,i-1}^{j}\right). \quad \text{(S3)}$$

Here, a spatial grid size (Δa) is set to be 0.4 nm, and each grid node is indexed by a subscript i. A temporal grid size (Δt) is set to be 10 fs, and a temporal evolution is indicated by a superscript j. We consider also a thermal boundary conductance which plays an important role in a thermal diffusion in the superlattice system. At the interface between SRO and STO, a boundary condition is given by the Fourier's law

$$q_{i+0.5}^{j} = \sigma_B (T_{i+1} - T_i), \quad \text{(S4)}$$

where q is a heat flux and σ_B is a thermal boundary conductance. At the surface, we simply choose the Neumann boundary condition.

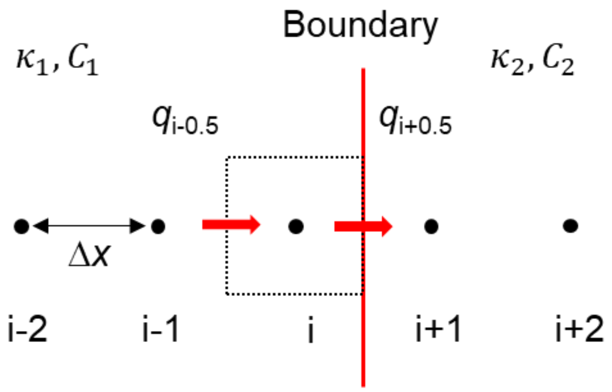

Figure S4. Schematic of the finite difference method for the two-temperature model analysis. Heat flux (q) is calculated by two adjacent grid points. We also consider the thermal boundary conductance between different thermal media.

S4. Sensitivity analysis of fitting parameters used in the two-temperature model

A transient reflectivity change is described by three different processes, namely (i) electron-phonon thermalization, (ii) thermalization between SRO-STO superlattice layers (SL thermalization), and (iii) heat diffusion from superlattice to substrate (Fig. S5(a)). Each of these three processes is related to the electron-phonon coupling constant (G_{ep}), the thermal boundary conductance (TBC) and the thermal conductivity of SRO (κ_{SRO}), respectively. To figure out whether these parameters can be determined reliably from our experimental results, we perform the sensitivity analysis for each parameter[7]. Sensitivity function S_α of a fitting parameter α is given by

$$S_\alpha = \frac{\alpha}{Y}\frac{\partial Y}{\partial \alpha}. \tag{S5}$$

Here, Y is a fitted function which is the transient reflectivity in this work. And, S_α dictates how much the fitting function Y is influenced by the fitting parameter α. Figure S5(b) shows sensitivity plots for G_{ep}, TBC and κ_{SRO}, which are given as a function of a pump-probe delay time. In the case of the $[30|30]_2$ SL, all three parameters have a high sensitivity value, and each parameter has its own temporal range with the high sensitivity, namely, before 10 ps for G_{ep}, between 10 ps and 100 ps for TBC, and after 100 ps for κ_{SRO}. These are consistent with our original prediction and also the simulation results displayed in Fig. 2(a) in the main text. In the case of the $[4|4]_{15}$ SL, G_{ep} and TBC have the high sensitivity in the early temporal range, i.e., before and after 2 ps, respectively. However, the sensitivity to κ_{SRO} appears very small in most of the temporal range investigated. This implies that G_{ep} and TBC can be reliably determined for the $[4|6]_{10}$ SL whereas κ_{SRO} would be estimated with a large uncertainty.

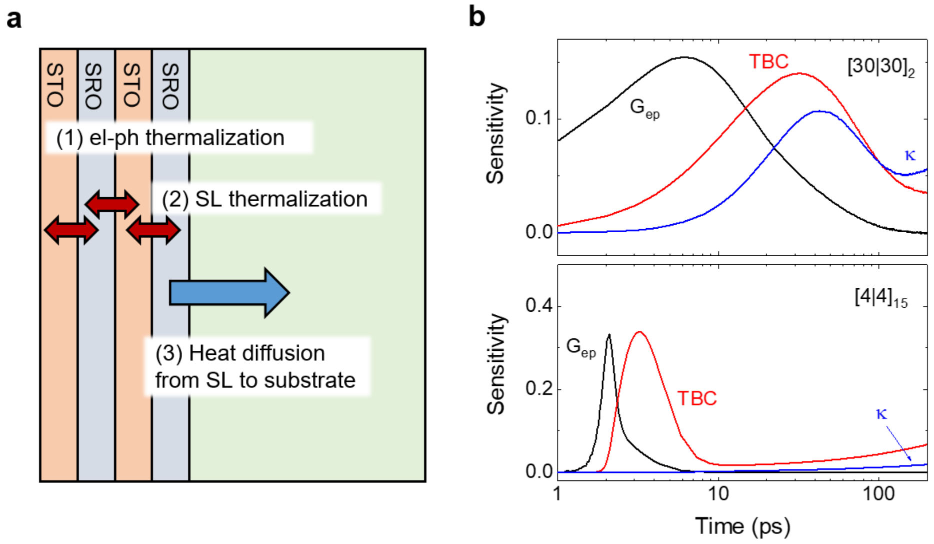

Figure S5. (a) Schematic of hot carrier energy relaxation process. Photo-excited electron transfers its energy to the lattice via an electron-phonon thermalization process, and the lattice is cooled by thermal diffusion. (b) Sensitivity plot for three fitting parameters of G_{ep}, TBC, and κ_{SRO} in the two-temperature model analysis for two SLs.

S5. Role of the electron-phonon thermalization in the hot carrier cooling process

The transient reflectivity becomes relaxed faster as the SRO layer becomes thinner, and this is attributed to the faster cooling of hot carriers. In understanding such results, although we have considered the contribution of the electron-phonon thermalization process, one may think about a possibility of the contribution of the thermal diffusion which would work more efficiently in a thinner layer of metal[8]. Indeed, the transient reflectivity change in $SrVO_3/SrTiO_3$ SLs could be explained by considering only the thermal diffusion without the electron-phonon thermalization[9].

In SRO/STO SLs, however, we confirm that the electron-phonon thermalization process is essential in understanding the hot electron cooling process. As shown in Fig.S6, if electron and lattice sub-systems would be immediately thermalized (G_{ep} = inf.) after the photoexcitation, although the response after 2 ps can be reasonably fitted with the contribution of the thermal diffusion, an initial fast decay after pumping cannot be explained. Indeed, RMS error which is a difference between experiment and fitting results is larger before 2 ps when we ignore the electron-phonon thermalization (Fig. S4(b)).

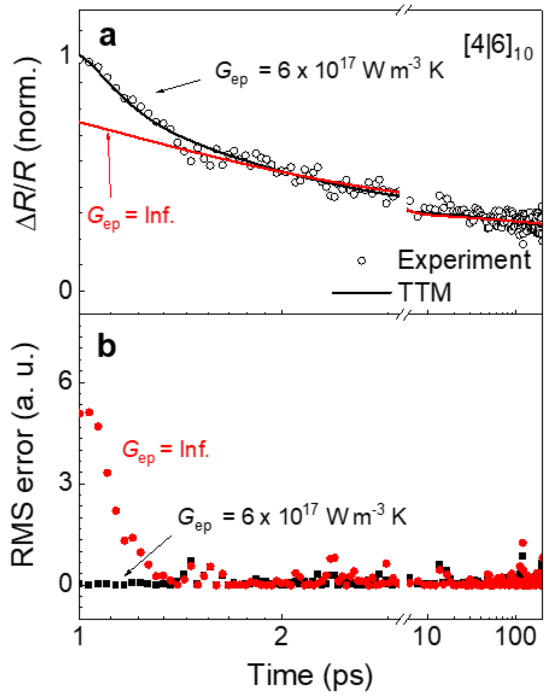

Figure S6. A contribution of the electron-phonon thermalization for the hot carrier energy relaxation process. **(a)** Experimental result of the $[4|6]_{10}$ SL and the TTM results obtained with two different values of G_{ep}. If we do not consider the electron-phonon thermalization, or, equivalently, consider only the thermal diffusion, the experiment results cannot be explained particularly in the initial stage after the photoexcitation. **(b)** RMS error of the TTM analyses.

S6. The two-temperature model analyses results

Figure S7 shows TTM fitting results for six representative experimental results of SRO single-films and SRO/STO SLs. Red and blue solid lines represent contributions from electron temperature (ΔT_e) and lattice temperature (ΔT_l), respectively. Successful fits to the experimental results clearly demonstrate that the hot electron temperature decreases faster for the films or superlattices with a thinner SRO layer. For the 125 uc thick single-film, $\Delta R/R$ rises exceptionally slowly, and it is attributed to the small el-ph coupling which gives rise to the slow increase in ΔT_l. A contribution from ΔT_e is missing (dashed line), and its reason is not clear at the moment.

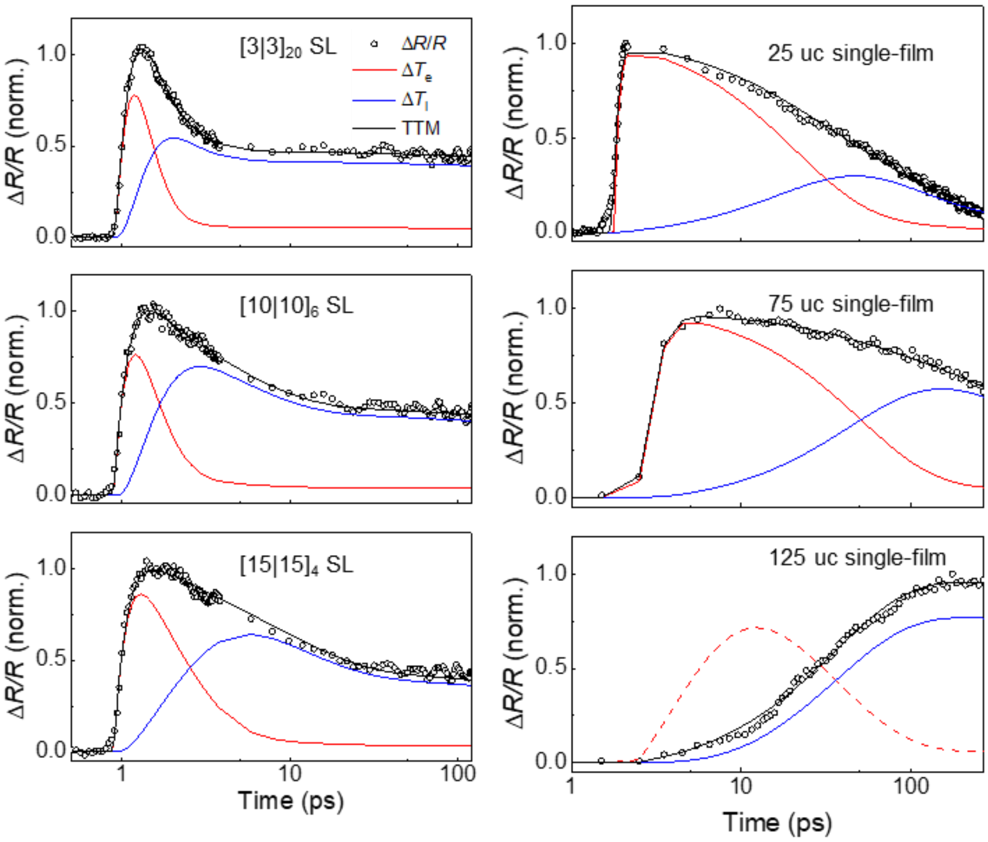

Figure S7. TTM fitting results of SRO thin film and superlattices with various thicknesses.

S7. Pump-power-dependent hot carrier cooling time

The electron-phonon coupling constant G_{ep} may be given differently when an initial electron temperature is extremely high[10]. In our two-temperature model analysis, however, we assume that the hot carrier temperature is not high enough to have such a nonlinear effect. In the two-temperature model, the relaxation time τ_{ep} of the hot carrier temperature via the electron-phonon thermalization is given as[11]

$$\tau_{ep} = \frac{\gamma_e (T_e + T_i)}{2G_{ep}}. \quad (S6)$$

Here, T_i is an initial temperature. Therefore, provided that G_{ep} remains the same, τ_{ep} should be in proportion to the pump power as it linearly increases T_e.

Figure S8 displays how the optical response changes as the pump power varies. An amplitude of the transient reflectivity increases linearly in proportion to the pump power for both the 25 uc single-film and the $[30|30]_2$ SL. This behavior is simply attributed to the increases in T_e and T_l with an increase of the pump power. Importantly, τ_{ep} increases also linearly in proportion to the pump power. This power-dependent relaxation time is in good agreement with the two-temperature model (Eq. S6), and implies that our experiment condition is in the linear regime where the electron-phonon coupling constant G_{ep} is uniquely defined.

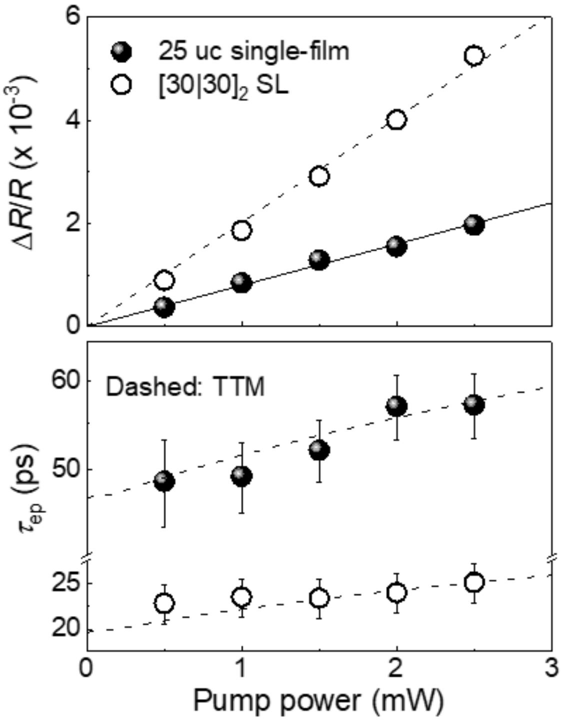

Figure S8. Pump-power-dependent electron-phonon thermalization time (τ_{ep}). τ_{ep} increases in proportion to the pump power, and this behavior is well explained by the two-temperature model (TTM).

S8. State-filling effect

An interpretation of the reflectivity change right after the photo-excitation is complicated due to an existence of non-thermalized photo-carriers which can have contributions to polarization grating, state-filling, anisotropic distribution, and so on[12]. Among them, we figure out the state-filling effect explicitly and analyze our results by separating out such contribution.

To extract the state-filling effect, we designed two different situations having an energy relationship of pump and probe beams, namely, $E_{pump}>E_{probe}$ and $E_{pump}<E_{probe}$ as depicted in Fig. S9(a). When $E_{pump}>E_{probe}$, the probe beam can respond to a rapid relaxation of non-thermalized carriers into the band edge; as the optical transition is less allowed due to the Pauli exclusion principle, the reflectivity of probe beam shows a drastic change. In particular, such state-filling effect will disappear as non-thermalize carriers become thermalized carriers via an optical phonon scattering and a carrier-carrier scattering[13]. When $E_{pump}<E_{probe}$, this process will not appear.

Figure S9(b) shows the transient reflectivity change results obtained for two different cases of $E_{pump}>E_{probe}$ and $E_{pump}<E_{probe}$. Here, the pump and probe energies are set to be 1.58 eV and 1.52 eV, respectively, for the first case, and the other way round for the second case. As expected, the first sharp peak is only observed in $E_{pump}>E_{probe}$ case, and it implies that the fast relaxation right after the photo-excitation is the state-filling effect rather than free carrier response. It should be noted that we have performed most of the pump-probe experiments in the condition of $E_{pump}<E_{probe}$, and have considered the state-filling effect explicitly in the other condition.

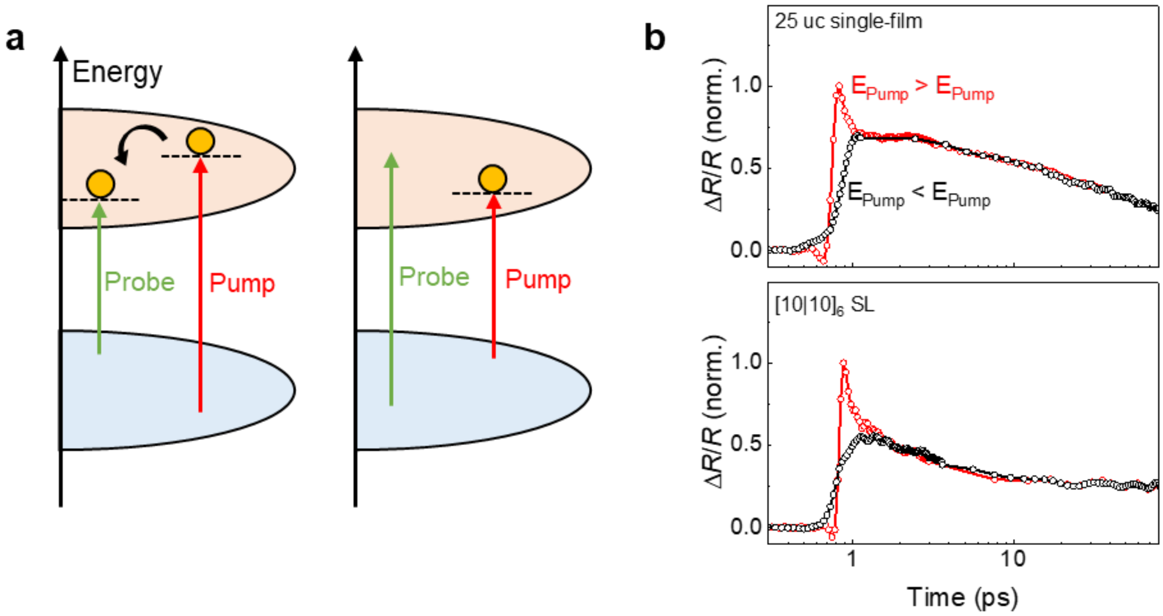

Figure S9. State-filling effect in SRO single-film and SRO/STO superlattice. Initial fast decay disappears when the energy of probe beam is larger than that of pump beam.

S9. Substrate effect for electron-phonon coupling in $SrRuO_3$ superlattices

Figure S10(a) shows transient reflectivity changes of the SRO/STO superlattices prepared on various substrates. Transient reflectivity increased right after pumping rapidly drops on a similar timescale for all the cases. After 5 ps, the slope of reflectivity change is slightly different depending on the substrate used, and it is attributed to different thermal conductivity values of substrates.

Figure S10(b) displays G_{ep} obtained from the TTM analysis. Indeed, there is no correlation between electron-phonon coupling and the substrate species. This result implies that the substrate or its phonon does not have a meaningful influence on G_{ep} of the SRO layer. Furthermore, although an electronic structure of SRO layers can be affected by a strain[14], such an influence is not visible even with a large variation of compressive or tensile strain measured with a lattice mismatch ranging from -4 % (compressive) to 2 % (tensile).

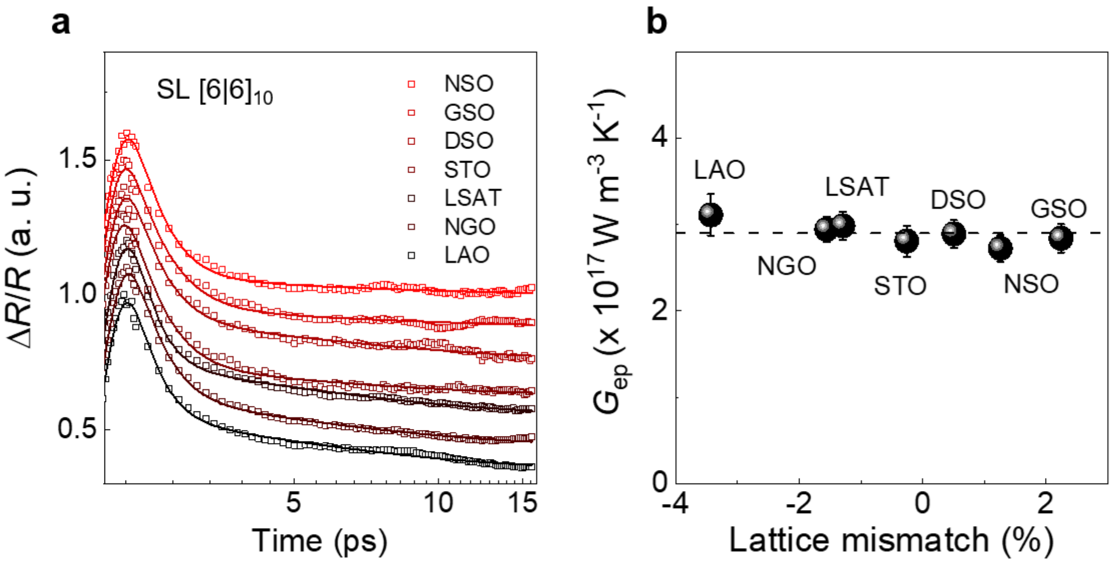

Figure S10. Electron-phonon coupling strength G_{ep} of SRO SLs grown for various substrates. There is no clear dependence on kinds of substrates even if the strain is largely changed.

S10. Temperature-dependent electron-phonon coupling

In the electron-lattice non-equilibrium state, the heat equation is described by

$$C_e \frac{dT_e}{dt} = -H(T_e, T_l). \tag{S7}$$

Here, C_e is an electron specific heat, and H is an electron-to-lattice energy transfer rate per unit volume which is given as a function of an electron temperature T_e and a lattice temperature T_l. As in conventional metals, an interaction between electron and acoustic phonons is first considered for the energy transfer process. In the two-temperature model, the energy transfer rate is described in a more detail as[11]

$$H(T_e, T_l) = U(T_e) - U(T_l) = G_{ep}(T)(T_e - T_l), \tag{S8}$$

$$U(T) = g_\infty T_D \left(\frac{T}{T_D}\right)^5 \int_0^{T_D/T} \frac{x^4}{e^x - 1} \mathrm{dx}. \tag{S9}$$

Here, g_∞ is an intrinsic electron-phonon coupling constant, and T_D is the Debye temperature. Temperature-dependent electron-phonon coupling constant $G_{ep}(T)$ is simply given by the first derivative of energy change rate, $U(T)$.

If the electron would be coupled with optical phonons, the modification should be made for Eq. (S9) about the energy change rate $U(T)$ as

$$U_{\mathrm{op}}(T) = -g_\infty \frac{T_D}{e^{\beta\hbar\omega_{\mathrm{op}}} - 1}. \tag{S10}$$

Here, the optical phonon is assumed to have no dispersion near the zone center. In this case, unlike the interaction with acoustic phonons, the energy change rate exponentially decreases as temperature decreases.

S11. Electron-phonon coupling strengths of various metals

Figure S11 summarizes the electron-phonon coupling strength of SRO films investigated in this work together with various noble metals. For noble metals, G_{ep} shows an increase in proportion to C_e/T. This general trend can be naturally understood as the higher electron density of states at the Fermi level gives rise to the larger el-ph coupling strength. For SRO films, however, the relationship between G_{ep} and C_e/T are largely different; although the SRO films in the thin film limit seem to show the general trend of noble metals, the major G_{ep} enhancement starting from the bulk SRO largely deviates from such trend. This implies that the large G_{ep} variations observed in SRO films should be understood by considering not only the electron density of states but also the variation in the phonon contribution as discussed in the main text.

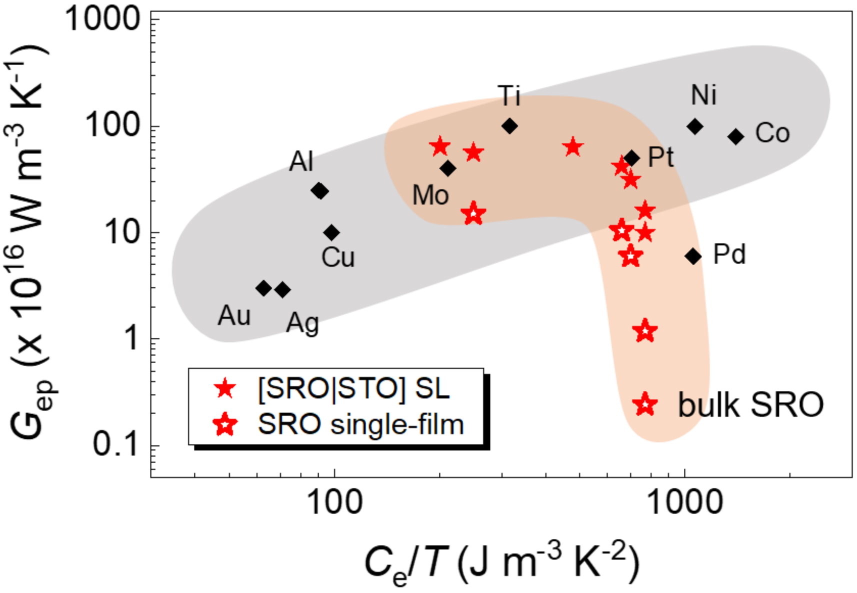

Figure S11. Electron-phonon coupling strength G_{ep} of SRO single-films and SLs. For comparison, the results for various noble metals are also included. G_{ep} is plotted versus C_e/T.

Reference

1 Seo, A., Stavropoulos, P. P., Kim, H. -H., Fursich, K., Souri, M., Connell, J. G., Gretarsson, H., Minola, M., Kee, H. Y. and Keimer, B. Compressive strain induced enhancement of exchange interaction and short-range magnetic order in Sr2IrO4 investigated by Raman spectroscopy. *Physical Reivew B* **100**, 165106 (2019).

2 Ordonez-Miranda, J., Yang, R. and Alvarado-Gil, J. J. Steady state and modulated heat conduction in layered systems predicted by the analytical solution of the phonon Boltzmann transport equation. *Journal of Applied Physics* **118**, 075103 (2015).

3 Jeong, S. G., Seo, A., Choi, W. S. . Atomistic Engineering of Phonons in Functional Oxide Heterostructures. *Advanced Science* **9** (2022).

4 Jeong, S. G., Min, T. W., Woo, S. M., Kim, J. W., Zhang, Y.-Q., Cho, S. W., Son, J. S., Kim, Y. M., Han, J. H., Park, S. K., Jeong, H. Y., Ohta, H., Lee, S., Noh, T. W., Lee, J. K. and Choi, W. S. . Phase Instability amid Dimensional Crossover in Artificial Oxide Crystal. *Physical Reivew Letter* **124**, 026401 (2020).

5 Collignon, C., Bourges, P., Fauqué, B. and Behnia K. . Heavy Nondegenerate Electrons in Doped Strontium Titanate. *Physical Reivew X* **10**, 031025 (2020).

6 Dehkordi, A. M., Bhattacharya, S., Darroudi, T.,Karakaya, M., Kucera, C., Ballato, J., Adebisi, R., Gladden, J. R., Podila, R., Rao, A. M., Alshareef, H. N. and Tritt, T. M. . Optimizing thermal conduction in bulk polycrystalline SrTiO3−δ ceramics via oxygen non-stoichiometry. *MRS Communications* **8**, 1470 (2018).

7 Koh, Y. K., Singer, S. L., Kim, W. C., Zide, J. M. O., Lu, H., Cahill, D. G., Majumdar, A. and Gossard, A. C. Comparison of the 3w method and time-domain thermoreflectance for measurements of the cross-plane thermal conductivity of epitaxial semiconductors. *Journal of Applied Physics* **105**, 054303 (2009).

8 Jiang, P., Qian, X. and Yang, R. Tutorial: Time-domain thermoreflectance (TDTR) for thermal property characterization of bulk and thin film materials featured. *Journal of Applied Physics* **124**, 161103 (2018).

9 Katsufuji, T., Saiki, T., Okubo, S., Katayama, Y. and Ueno, K. Thermal conductivity of SrVO3-SrTiO3 thin films: Evidence of intrinsic thermal resistance at the interface between oxide layers. *Physical Reivew Materials* **2**, 051002 (2018).

10 Li, Y. a. J., P. Ab initio calculation of electron temperature dependent electron heat capacity and electron-phonon coupling factor of noble metals. *Computational Materials Science* **202**, 110959 (2022).

11 Groeneveld, R. H. M. a. S., R. and Lagendijk, A. Femtosecond spectroscopy of electron-electron and electron-phonon energy relaxation in Ag and Au. *Physical Reivew B* **51**, 17 (1995).

12 Sabbah, A. J. a. R., D. M. Femtosecond pump-probe reflectivity study of silicon carrier dynamics. *Physical Reivew B* **66**, 165217 (2002).

13 Fatti, N. D., Langot, P., Tommasi, R. and Vallée, F. . Temperature-dependent electron-lattice

thermalization in GaAs. *Physical Reiwew B* **59**, 4576 (1999).

14 Tian, D., Liu, Z. and Shen, S. Manipulating Berry curvature of SrRuO3 thin films via epitaxial strain. *PNAS* **118**, 18 (2021).
